# Two-site study on performances of a commercially available MALDI-TOF MS-based assay for the detection of colistin resistance in *Escherichia coli*

**DOI:** 10.1007/s10096-023-04587-9

**Published:** 2023-03-27

**Authors:** Gerald Larrouy-Maumus, Laurent Dortet, Ilka D. Nix, Thomas Maier, Boris Oberheitmann, Katrin Sparbier, Markus Kostrzewa

**Affiliations:** 1 Centre for Bacterial Resistance Biology, Department of Life Sciences, Imperial College London, London, SW7 2AZ UK; 2 Department of Bacteriology-Hygiene, Bicêtre Hospital, Assistance Publique-Hôpitaux de Paris, Le Kremlin-Bicêtre, France; 3 Bruker Daltonics GmbH & Co KG, Bremen, Germany

**Keywords:** MALDI, Lipid A, *E. coli*, Polymyxins, Diagnostics

## Abstract

Colistin is a last resort drug for the treatment of multiple drug-resistant (MDR) Gram-negative bacterial infections. Rapid methods to detect resistance are highly desirable. Here, we evaluated the performance of a commercially available MALDI-TOF MS-based assay for colistin resistance testing in *Escherichia coli* at two different sites. Ninety clinical *E. coli* isolates were provided by France and tested in Germany and UK using a MALDI-TOF MS-based colistin resistance assay. Lipid A molecules of the bacterial cell membrane were extracted using the MBT Lipid Xtract Kit™ (RUO; Bruker Daltonics, Germany). Spectra acquisition and evaluation were performed by the MBT HT LipidART Module of MBT Compass HT (RUO; Bruker Daltonics) on a MALDI Biotyper® sirius system (Bruker Daltonics) in negative ion mode. Phenotypic colistin resistance was determined by broth microdilution (MICRONAUT MIC-Strip Colistin, Bruker Daltonics) and used as a reference. Comparing the results of the MALDI-TOF MS-based colistin resistance assay with the data of the phenotypic reference method for the UK, sensitivity and specificity for the detection of colistin resistance were 97.1% (33/34) and 96.4% (53/55), respectively. Germany showed 97.1% (33/34) sensitivity and 100% (55/55) specificity for the detection of colistin resistance by MALDI-TOF MS. Applying the MBT Lipid Xtract™ Kit in combination with MALDI-TOF MS and dedicated software showed excellent performances for *E. coli*. Analytical and clinical validation studies must be performed to demonstrate the performance of the method as a diagnostic tool.

## Introduction

Antimicrobial resistance is an issue of global importance and one of the defining public health concerns of our time [1]. The limited pipeline of novel antimicrobials and the spread of multidrug-resistant (MDR) organisms have increased our reliance on a few last-line antibiotics for the treatment of MDR Gram-negative bacteria*.* Crucial last-resort agents are the polymyxin antibiotics, such as polymyxin B and colistin [2, 3].

In Gram-negative bacteria like *Escherichia coli*, polymyxin resistance mostly occurs as a consequence of lipopolysaccharide (LPS) modifications, with the addition of phosphoethanolamine (pEtN) and/or 4-amino-L-arabinose (L-Ara4N) cationic groups on the lipid A portion of LPS [4, 5]. These lipid A modifications often arise due to (i) alterations of the PmrAB and PhoPQ two-component systems and mutations of the negative regulator of PhoPQ and MgrB that are both chromosome-encoded or (ii) because of the activity of a plasmid-borne pEtN transferase called mobile colistin resistance (MCR) enzyme [6]. The first MCR enzyme, *mcr-1*, was reported in 2016 [7], and this discovery was followed by the rapid identification of other mobile polymyxin resistance genes [8–11].

We have previously reported on the development of the MALDIxin test, a diagnostic tool based on matrix-assisted laser desorption/ionization-time of flight (MALDI-TOF) mass spectrometry (MS) that can be used to detect colistin resistance using whole bacteria [12–18]. Although fast and effective, this test was not optimized for routine use in diagnostic microbiology laboratories. The main limitations were that such an approach was not developed for the MALDI-TOF mass spectrometers widely used for bacterial identification in clinical microbiology laboratories, as well as not having a robust and easy standardized kit and software module to perform automated data analysis and interpretation. In addition, the standard workflow of the MALDIxin test required centrifugation steps [12, 13, 16–18], the use of either super-2,5-Dihydroxybenzoic acid or Norharmane matrix, which led to the non-homogenous co-crystallization of the biological sample with the matrix on the MALDI target plate, precluding any automation of the data acquisition and processing.

The aim of this study was to determine the performances and reproducibility of a standardized methodology designed (and commercialized) for the low-resolution linear MALDI-TOF MS instrument in negative ion mode employed by the MALDI Biotyper® sirius system (Bruker Daltonics) to accurately identify colistin resistance in clinical *E. coli* isolates by detecting addition of both pEtN and L-Ara4N moieties to lipid A and reporting the results using an automated software module.

## Materials and methods

### Bacterial strains

A collection of 90 *E. coli* clinical isolates (Table 1) was used in this study which was provided by the Centre National de Reference associé Résistance aux Antibiotiques “Entérobactéries productrices de carbapénèmases.” The same sets of strains were sent as heat-inactivated isolates to Imperial College London, UK, and Bruker Daltonics GmbH & Co KG, Bremen, Germany, for the evaluation of the MBT Lipid Xtract™ Kit (RUO, Bruker Daltonics) in combination with the MBT HT LipidART Module (RUO, Bruker Daltonics). Evaluation centers in the UK and Germany were blinded of the colistin resistance phenotype and of the colistin resistance mechanisms. Preliminary experiments showed comparable results of lipid A extracts of heat-inactivated isolates and viable strains using the MBT Lipid Xtract™ Kit in conjunction with the MBT HT LipidART Module (data not shown).

**Table 1:** Colistin-resistant and colistin-susceptible clinical *E. coli* strains used in this study

Strain-ID	Resistance mechanism to polymyxins			Acquired resistance to β-lactams^e^	Country (and town) of isolation	Colistin resistance tested by LipidART			
	Genotypic characterization^a^	Phenotypic characterization				UK		Germany	
		MIC to colistin^b^	SIR categorization^d^			S / R	Modification	S / R	Modification
Ecoli_R01	Mutations in PmrB (D14N, S71C + V83A)	8	R	None	France	R	pEtN + Ara4N	R	pEtN + Ara4N
Ecoli_R02	*mcr-1*	4	R	None	France	R	pEtN	R	pEtN
Ecoli_R03	*mcr-1*	2	S	None	France (Clermont-Ferrand)	S	–	S	–
Ecoli_R05	*mcr-1*	4	R	OXA-48	Belgium	R	pEtN	R	pEtN
Ecoli_R06	*mcr-1*	4	R	OXA-48	Belgium	R	pEtN	R	pEtN
Ecoli_R07	Unknown	8	R	None	Belgium	R	pEtN + Ara4N	R	pEtN + Ara4N
Ecoli_R08	Unknown	4	R	Penicillinase	Belgium	R	pEtN + Ara4N	R	Ara4N
Ecoli_R09	*mcr-1*	8	R	NDM-1	Belgium	R	pEtN	R	pEtN
Ecoli_R10	–	0.5^c^	S	KPC-28 + OXA-48	France (Clermont-Ferrand)	S	–	S	–
Ecoli_R11	*mcr-2*	4	R	None	Portugal	R	pEtN	R	pEtN
Ecoli_R12	*mcr-1*	4	R	SHV-12	Belgium	R	pEtN	R	pEtN
Ecoli_R13	*mcr-1*	4	R	CTX-M-15	France (Grenoble)	R	pEtN	R	pEtN
Ecoli_R14	*mcr-1*	0.25	S	KPC-28 + OXA-48	France (Clermont-Ferrand)	S	–	S	–
Ecoli_R15	*mcr-1*	8	R	TEM-15	France (Wissous)	R	pEtN	R	pEtN
Ecoli_R16	Mutation in PmrB (G160E)	8	R	None	France (Bordeaux)	R	pEtN + Ara4N	R	pEtN + Ara4N
Ecoli_R17	*mcr-1*	8	R	CTX-M-15 + SHV-12 + TEM-1	France (Le Mans)	R	pEtN	R	pEtN
Ecoli_R18	*mcr-1*	8^c^	R	CTX-M-1 + TEM-1	France (Rodez)	R	pEtN	R	pEtN
Ecoli_R31	*mcr-1*	4	R	CTX-M-2	Argentina	R	pEtN	R	pEtN
Ecoli_R32	*mcr-1*	8^c^	R	TEM-1B	Argentina	R	pEtN	R	pEtN
Ecoli_R33	*mcr-1*	4	R	CTX-M-2	Argentina	R	pEtN	R	pEtN
Ecoli_R34	*mcr-1.5*	4	R	TEM-1B	Argentina	R	pEtN	R	pEtN
Ecoli_R35	*mcr-1*	4	R	None	Argentina	R	pEtN	R	pEtN
Ecoli_R36	*mcr-1.5*	− ^c^	NA	CTX-M-2	Argentina	R	pEtN	R	pEtN
Ecoli_R37	*mcr-3.2*	8	R	CTX-M-55	France	R	pEtN	R	pEtN
Ecoli_R38	Unknown	8	R	TEM-1 + SHV-12	France	R	pEtN + Ara4N	R	pEtN + Ara4N
Ecoli_R39	Unknown	2	S	None	France	S	–	S	–
Ecoli_R40	Unknown	16	R	None	France	R	Ara4N	R	pEtN + Ara4N
Ecoli_R41	Unknown	32	R	CTX-M-27	France	R	Ara4N	R	pEtN + Ara4N
Ecoli_R42	Unknown	32	R	None	France	R	pEtN + Ara4N	R	pEtN + Ara4N
Ecoli_R43	Unknown	0.5^c^	S	None	France	S	–	S	–
Ecoli_R44	Unknown	64	R	CTX-M-1	France	R	pEtN + Ara4N	R	Ara4N
Ecoli_R45	Unknown	4	R	None	France	R	pEtN + Ara4N	R	Ara4N
Ecoli_R46	Unknown	4	R	None	France	R	pEtN + Ara4N	R	pEtN + Ara4N
Ecoli_R47	Unknown	16	R	CTX-M-15 + OXA-1	France	R	pEtN + Ara4N	R	Ara4N
Ecoli_R48	*mcr-1*	4	R	None	France (Paris)	R	pEtN	R	pEtN
Ecoli_R51	–	0.5	S	NDM-5	France (Aulnay)	S	–	S	–
Ecoli_R52	–	0.5	S	NDM-5	France (Vierzon)	S	–	S	–
Ecoli_R53	–	0.5	S	OXA-48	France (Strasbourg)	S	–	S	–
Ecoli_R54	Unknown	8	R	ESBL	France (Toulouse)	R	pEtN + Ara4N	R	Ara4N
Ecoli_R55	Unknown	4	R	ESBL	France (Wissous)	R	pEtN	R	pEtN
Ecoli_R56	–	0.5^c^	S	NDM-1	France (Rang du Fliers)	S	–	S	–
Ecoli_R57	Unknown	16	R	None	France (Lyon)	R	pEtN + Ara4N	R	pEtN + Ara4N
Ecoli_R58	Unknown	8	R	None	France (Lyon)	R	Ara4N	R	pEtN + Ara4N
Ecoli_R59	–	0.25^c^	S	OXA-48	France (Créteil)	R	Ara4N	S	–
Ecoli_R60	–	0.5	S	VIM-1	France (Marseille)	S	–	S	–
Ecoli_R61	–	0.25	S	NDM-1	France (Clermont-Ferrand)	S	–	S	–
Ecoli_S01	–	0.25	S	None	Laboratory strain *E. coli* J53	S	–	S	–
Ecoli_S02	–	0.5	S	None	Laboratory strain *E. coli* TOP10	S	–	S	–
Ecoli_S03	–	0.25	S	None	France (Le Kremlin-Bicêtre)	S	–	S	–
Ecoli_S04	Unknown	8	R	None	France (Le Kremlin-Bicêtre)	S	–	S	–
Ecoli_S05	–	0.25	S	None	France (Le Kremlin-Bicêtre)	S	–	S	–
Ecoli_S06	–	0.5	S	None	France (Le Kremlin-Bicêtre)	S	–	S	–
Ecoli_S07	–	0.125	S	None	France (Le Kremlin-Bicêtre)	S	–	S	–
Ecoli_S08	–	2	S	None	France (Le Kremlin-Bicêtre)	S	–	S	–
Ecoli_S09	–	0.25	S	None	France (Le Kremlin-Bicêtre)	S	–	S	–
Ecoli_S10	–	0.25	S	None	France (Le Kremlin-Bicêtre)	S	–	S	–
Ecoli_S11	–	0.25	S	None	France (Le Kremlin-Bicêtre)	S	–	S	–
Ecoli_S12	–	0.5	S	None	France (Le Kremlin-Bicêtre)	S	–	S	–
Ecoli_S13	–	0.5	S	Penicillinase	France (Le Kremlin-Bicêtre)	S	–	S	–
Ecoli_S14	–	0.25	S	Penicillinase	France (Le Kremlin-Bicêtre)	S	–	S	–
Ecoli_S15	–	0.5	S	Penicillinase	France (Le Kremlin-Bicêtre)	S	–	S	–
Ecoli_S16	–	0.25	S	Penicillinase	France (Le Kremlin-Bicêtre)	S	–	S	–
Ecoli_S17	–	0.25	S	Penicillinase	France (Le Kremlin-Bicêtre)	S	–	S	–
Ecoli_S18	–	0.25	S	Penicillinase	France (Le Kremlin-Bicêtre) ^f^	S	–	S	–
Ecoli_S19	–	0.25	S	Penicillinase	France (Le Kremlin-Bicêtre)	S	–	S	–
Ecoli_S20	–	0.25	S	Penicillinase	France (Le Kremlin-Bicêtre)	R	Ara4N	S	–
Ecoli_S21	–	0.25	S	Penicillinase	France (Le Kremlin-Bicêtre)	S	–	S	–
Ecoli_S22	–	0.25	S	Penicillinase	France (Le Kremlin-Bicêtre)	S	–	S	–
Ecoli_S23	–	0.25	S	CTX-M-15	France	S	–	S	–
Ecoli_S24	–	0.25	S	CTX-M-1	France	S	–	S	–
Ecoli_S25	–	0.5	S	CTX-M-3	France	S	–	S	–
Ecoli_S26	–	0.5	S	CTX-M-14	France	S	–	S	–
Ecoli_S27	–	0.5	S	CTX-M-14	France	S	–	S	–
Ecoli_S28	–	0.25	S	CTX-M-15	France	S	–	S	–
Ecoli_S29	–	0.25	S	CTX-M-15	France	S	–	S	–
Ecoli_S30	–	0.5	S	SHV-2a	France	S	–	S	–
Ecoli_S31	–	0.25	S	SHV-12	France	S	–	S	–
Ecoli_S32	–	0.25	S	TEM-3	France	S	–	S	–
Ecoli_S33	–	1	S	TEM-24	France	S	–	S	–
Ecoli_S34	–	0.25	S	KPC-2	France	S	–	S	–
Ecoli_S35	–	2	S	NDM-1 + OXA-1 + TEM-1	France	S	–	S	–
Ecoli_S36	–	0.25	S	NDM-4 + CTX-M-15 + OXA-1	France	S	–	S	–
Ecoli_S37	–	0.25	S	NDM-5+ TEM-1 + CTX-M-15	France	S		S	–
Ecoli_S38	–	0.25	S	VIM-1	France	S		S	–
Ecoli_S39	–	0.5	S	IMP-8 + SHV-12	France	S		S	–
Ecoli_S40	–	0.125	S	OXA-48 + CTX-M-15	France	S		S	–
Ecoli_S41	–	0.5	S	OXA-244	France	S		S	–
Ecoli_S42	–	0.5	S	OXA-181	France	S		S	–
Ecoli_S43	–	0.25	S	OXA-204 + CMY-4 + CTX-M-15	France	S		S	–
Ecoli_S44	–	0.25	S	None	Laboratory strain *E. coli* DH5a	S		S	–

^a^For unknown mechanisms, the absence of mutations in *mgrB*, *pmrA*, *pmrB*, *phoP*, and *phoQ* have been checked by PCR and sequencing, and the strain was negative for *mcr-*like genes. Regarding *mcr* variants, the presence of *mcr-1* to *mcr-10* has been checked by PCR and sequencing of the amplicon in the case of a positive signal

^b^MIC, minimal inhibitory concentration, tested by broth microdilution. EUCAST breakpoint 2 µg/ml, according to valid breakpoint tables V. 12.0 of The European Committee on Antimicrobial Susceptibility Testing

^c^Median result for triplicate testing of broth microdilution

^d^S, susceptible; R, resistant; NA, not applicable

^e^Carbapenemases are shown in bold, and extended spectrum β-lactamases are underlined

^f^Strains from Le Kremlin-Bicêtre were isolated in 2016 and 2017 from different patients and hospitalized in different wards at different date

### Genotype determination

Identification of commonly encountered *mcr* genes (*mcr-1 to mcr-10)* was performed by PCR and sequencing of the amplicon in case of a positive signal (Table 1) [19]. For colistin-resistant isolates that were negative for the tested *mcr* genes, mutations in *mgrB*, *pmrA*, *pmrB*, *phoP*, and *phoQ* have been checked by PCR and sequencing. The β-lactamase encoding genes were identified as previously described [20].

### Susceptibility testing

Broth microdilution was performed as a phenotypic reference method using MICRONAUT MIC-Strip Colistin (Bruker Daltonics), following the manufacturer’s instructions. MICRONAUT MIC-Strip Colistin is a validated IVD product according to DIN EN ISO 20776- 2 [21] and was tested by EUCAST showing 99% essential agreement [22]. Classification of colistin susceptibility or resistance was done according to EUCAST breakpoints (version 12.0) [23]. In case of discrepancies between the MALDI-TOF MS-based result and classification result of the reference method, broth microdilution was repeated in triplicate, and the median result was used as the final reference result.

### Extraction of lipid A

Lipid A from *E. coli* was extracted using the MBT Lipid Xtract™ Kit, following the manufacturer’s instructions. Briefly, bacteria were grown overnight in the Mueller–Hinton agar, the equivalent of a 1-μL inoculation loop placed into a 1.5-mL low-binding microtube, and mixed in 50 μL of MBT Lipid Xtract Hydrolysis buffer. A total of 44 μL of the cell suspension was discarded, and the remaining 6 μL was submitted with the lid of the tube closed to a heating process at 90 °C for 10 min. The tubes were left for 2 min with the lid open to completely evaporate the buffer. The dried pellets were washed with 50 μL of MBT Lipid Xtract washing buffer for a few seconds without dissolving the pellet. The total volume of the washing buffer was discarded by pipetting. Finally, 5 μL of the matrix was pipetted up and down for 15–20 s to resuspend the dried pellet, and 2 μL of the suspension was spotted onto either an MSP 96 polished steel target (Bruker Daltonics, Part-No. 8280800) or an MBT Biotarget 96 (Bruker Daltonics, Part-No. 1840375).

### Acquisition of MALDI-TOF mass spectra and data analysis

The spectra were recorded in the linear negative-ion mode (laser intensity 45%, ion source 1 = 15.00 kV) of the MALDI Biotyper® sirius system. Each spectrum corresponded to ion accumulation of 200–1000 laser shots randomly distributed on the spot.

Spectra acquisition and simultaneous evaluation were done automatically in the MBT Compass HT using the MBT HT LipidART Module. A “log[SN](R)” value is calculated by the software. The log [SN](R) contains two important information of the lipid A mass spectra. The first value is an indicator of the peak intensities/signal-to-noise values (SN) of detected lipid A molecules. The second value shows the ratio of the lipid A modification to the native lipid A peak. The log [SN](R) value shall be used as an indicator of the general spectra quality and the height of the modified lipid A peaks compared to the native lipid A peak.

For visual inspection, the spectra obtained were processed with default parameters using FlexAnalysis v.3.4 software (Bruker Daltonics, Germany).

Performance evaluation was done according to DIN EN ISO 20776–2:2021 [21].

## Results and discussion

The MBT Lipid Xtract™ Kit was evaluated using a panel of 90 *E. coli* clinical isolates (Table 1). Using broth microdilution as the phenotypic reference method, 34 strains were tested as colistin resistant and 55 strains as colistin susceptible, respectively. One strain showed inconsistent MIC results and was defined as not applicable.

**Fig. 1:**
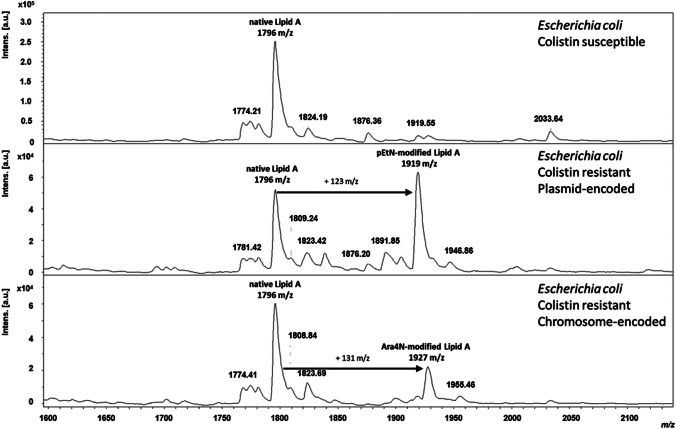
MALDI-TOF mass spectra of colistin susceptible and colistin resistant MCR-producing *E. coli.* Colistin* s*usceptible *E. coli* strain showed an unmodified native lipid A peak at 1796 m/*z* (hexa-acyl diphosphoryl lipid A). Colistin resistant *E. coli* strain showed an unmodified native lipid A peak at 1796 m/*z* and a pEtN-modified lipid A at 1919 m/*z* (plasmid-encoded) or an Ara4N-modified lipid A at 1927 m*/z* (chromosome-encoded)

For all colistin-susceptible *E. coli* clinical isolates in this panel, a single peak at *m/z* 1796.2 was detected, assigned to hexa-acyl diphosphoryl lipid A containing four C14:0 3-OH, one C14:0, and one C12:0, confirming that the lipid A in these strains was unmodified (Fig. 1). For colistin-resistant clinical isolate strains, both the native lipid A peak and the additional pEtN peak at *m*/*z* 1919.2 were observed, corresponding to the addition of one residue of pEtN (+ 123 mass unit) (Fig. 1) or the additional peak at *m*/*z* 1927.2 corresponding of the addition of one residue of L-Ara4N (+ 131 mass unit) at the 4′-phosphate of lipid A (Fig. 1).

Evaluation of the performance of the MALDI-TOF MS-based colistin resistance assay was done according to valid guidelines [21], and broth microdilution was used as the phenotypic reference method. The mass spectra of the 90 *E. Coli* clinical isolates were acquired in the negative ion mode on the MALDI Biotyper® sirius system. Using the MBT HT LipidART Module, the sensitivity and specificity of the method were 97.1 and 96.4%, respectively, for the UK site and 97.1 and 100%, respectively, for the German site (Table 2).

**Table 2:** Performance of the MALDI-TOF MS-based colistin resistance assay. Sensitivity and specificity were calculated for valid tests (clear result in the reference method)

	UK	Germany
Sensitivity (%)	97.1	97.1
Specificity (%)	96.4	100.0
Validity (%)	98.9	98.9
True positive, *n*	33	33
True negative, *n*	53	55
False positive, *n*	2	0
False negative, *n*	1	1
Not applicable, *n*	1	1
Total valid, *n*	89	89

For the German site, one false negative sample was observed (Ecoli_S04). The sample showed an MIC of 8 µg/mL but was identified as susceptible by the MALDI-TOF MS-based method. This discrepancy was not observed for the viable strain (data not shown).

At the UK site, the same sample was detected as false negative (Ecoli_S04), and two other samples were observed as false positive (E.coli_R59 and Ecoli_S20). The reference method showed a susceptible result, and the MALDI-TOF MS-based method revealed modified lipid A, which means colistin resistance due to calculated (*R*)-values that were close to the algorithm-based cutoff value.

Regarding the results, the strains, Ecoli_R59 and Ecoli_S20, show the discrepancy between the two sites. That could be due to the low presence of a non-specific peak at the m/z assigned to the L-Ara4N range and that the automated software pickup that background as positive or negative depending on the site, regarding the discrepancy between the two sites and whole genome sequencing data, which are Ecoli_R03, Ecoli_R10, Ecoli_R14, Ecoli_R39, Ecoli_R43, Ecoli_R51, Ecoli_R56, Ecoli_R59, Ecoli_R60, and Ecoli_R61. For strains like Ecoli R_14, one possibility is that *mcr-1* expression could be induced by the presence of colistin [24]. However, as no shift in MIC above 2 μg/mL was observed, it is likely that plasmid has been lost due to the long storage of the clinical isolates in the − 80 °C freezer and by freeze/thawing the stocks. We, therefore, recommend performing the assay as soon as possible after the isolation of the strains.

For strains like Ecoli_R03, even if carrying *mcr-1* and having a MIC at 2 μg/mL, which is at the breakpoint, no modification of the lipid A could be reported. However, the MIC and our assays are performed in vitro, and we cannot rule out that exposure to host factors could stimulate the expression of *mcr-1*, rendering the bacteria resistant to colistin. In another topic, for example, it has been reported that human serum induces antibiotic tolerance in *Staphylococcus aureus* by triggering the staphylococcal GraRS two-component system, resulting in increased peptidoglycan accumulation [25]. We cannot rule out that in the case of colistin resistance, host factors could possibly trigger the resistance in strains carrying mcr plasmids. That is why, in some cases, one could recommend taking into consideration strains that have an MIC of 2 μg/mL as resistant [26].

Overall, this study showed a high reproducibility of the MALDI-TOF MS-based colistin resistance assay revealing comparable results for both test sites (Table 2).

**Fig. 2:**
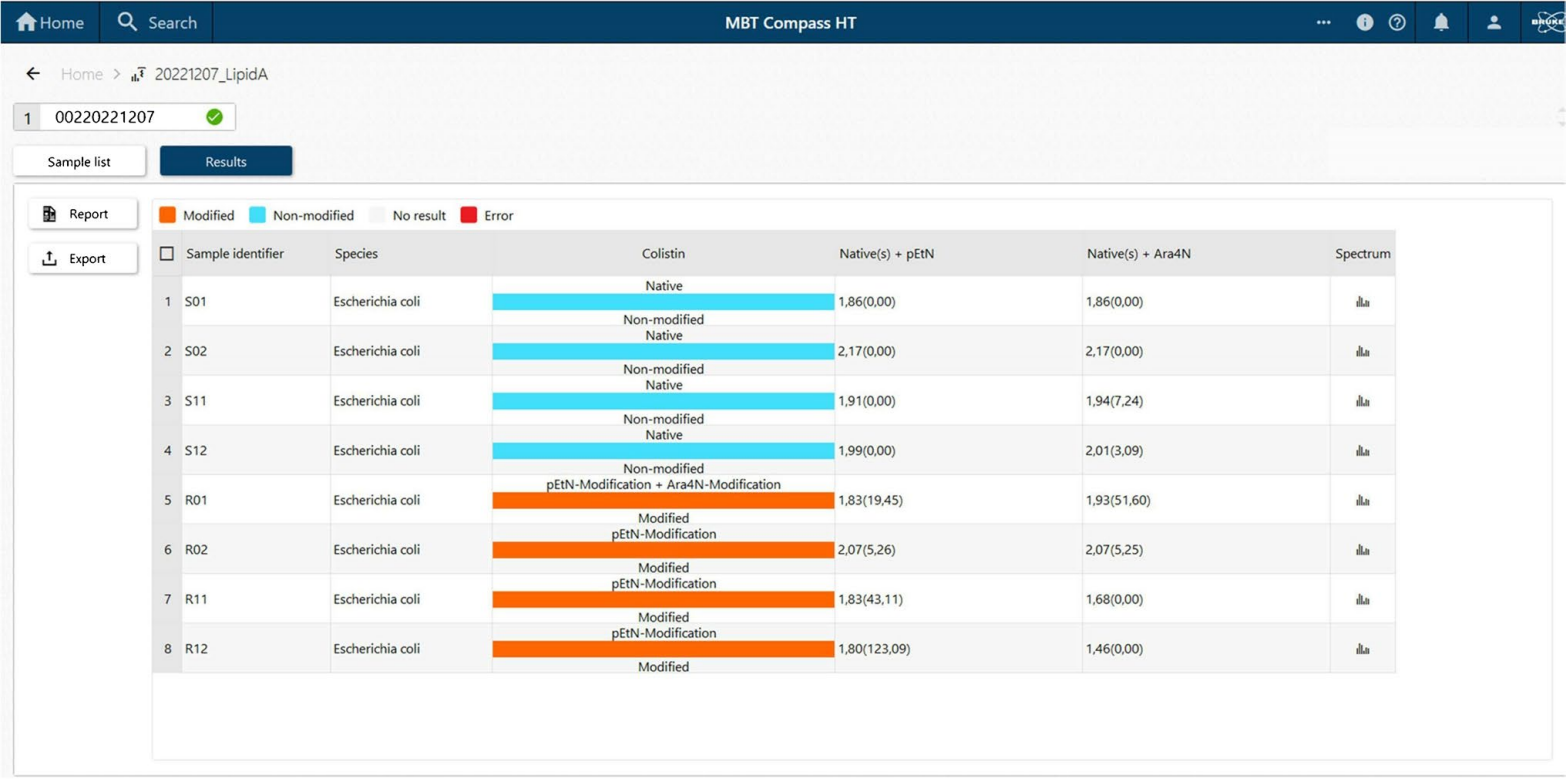
Result of the MBT HT LipidART Module in MBT Compass HT. The LipidART software module evaluates the acquired spectra and calculates log(SN)[R] values. The log(SN)[R] values are used to classify the spectra. If only the native lipid A molecule is detectable, the result is “native/non-modified.” If a modified lipid A variant is detectable in the spectrum, a “modified” result is obtained. Here, a differentiation between pEtN modification (+ 123 m*/z*) and Ara4N modification (+ 131 m*/z*) is possible

**Fig. 3:**
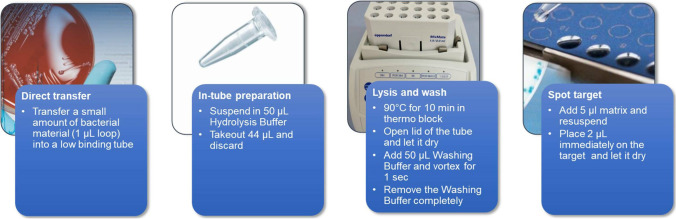
Workflow of the MBT Lipid Xtract™ Kit. One colony is transferred in a low-binding 1.5 mL microtube, 50 μL of hydrolysis buffer is added, the colony is suspended, and then, 44 μL are removed. The remaining 6 μL is placed into a thermo block set at 90 °C for 10 min. This hydrolysate is then dried by letting the tube open for 2 min. The microtube is subsequently placed on the bench at room temperature, 50 μL of washing buffer is added, and the solution is vortexed for 1 s. That washing step allows the removal of molecules that could interfere with the ionization of lipid A. Following that step, 50 μL of the washing buffer is removed. To the remaining material, 5 μL of the matrix is added, and the sample is resuspended. A total of 2 μL is immediately deposited onto the target. Once dried, the MALDI target plate is then placed into MALDI Biotyper sirius® system, and data are analyzed in the negative ion mode

**Fig. 4:**
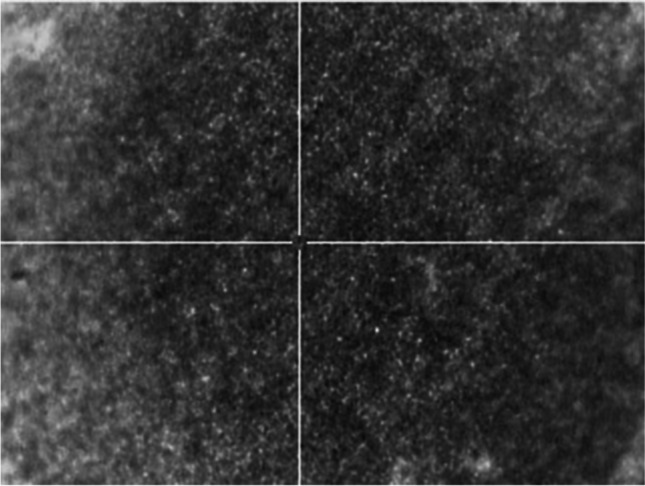
Homogeneous co-crystallization of lipid extract with matrix on an MBT Biotarget 96 after MBT Lipid Xtract™ Kit preparation

Previous studies suggested that the MALDIxin test was promising for the rapid identification of colistin resistance in Gram-negatives [12–18]. Unfortunately, this homemade version of the test was less suitable for use in routine diagnostics due to the requirement of the high amount of biological material and high laser power at the limit of the MALDI system (> 90%). The MALDI-TOF MS-based assay presented here requires very less biological material, no centrifugation step, and only short hands-on time. Additionally, the measurement can be performed with the same low-resolution settings used for other clinical applications on the MALDI Biotyper® sirius system. Furthermore, the development of a software module able to automatically interpret the MALDI-TOF spectrum is far more accurate for diagnostic use (Fig. 2). Compared to the MALDIxin test, the assay presented here is, therefore, more suitable for use in routine diagnostics (Fig. 3). Of note, with this new commercial kit, very homogeneous crystallization on the target surface can be achieved with the MBT Lipid Xtract matrix (Fig. 4) that is crucial for high reproducibility and automation of MALDI-TOF MS measurements.

After the appropriate development of in vitro diagnostic (IVD) consumables and software, following further clinical and analytical studies, it will be a very rapid and easy way to diagnose colistin resistance in clinical laboratories in the near future. Time-to-result will be significantly shortened compared to standard susceptibility testing methods in routine diagnostics. Preliminary studies showed that the method might be suitable for testing directly from positive blood cultures (data not shown) and would accelerate the diagnostic by at least one further day providing a same-day result.

This study has some limitations as it focused on *E. coli* colistin-resistant clinical isolates. Indeed, colistin resistance has also been reported in other Gram-negative bacteria such as *Klebsiella* spp., *Pseudomonas* spp., and *Acinetobacter baumannii*. Accordingly, this new approach should be expanded to those Gram-negative pathogens, too.

Overall, this study represents a major step towards the routine application of MALDI-TOF-based detection of colistin resistance and lays the foundations for a rapid diagnostic test for colistin resistance that will be readily accessible to many clinical microbiology laboratories. As such, it will facilitate improved management and treatment of patients with challenging MDR Gram-negative infections, particularly in countries where carbapenemase production is highly prevalent, leading to the increasing use of polymyxins, for which resistance emerged and started to disseminate.

## Data Availability

Data are available from the corresponding authors on reasonable request.
